# Supplementary material for: Environmental sampling for typhoidal Salmonellas in household and surface waters in Nepal identifies potential transmission pathways
Source: PLoS Negl Trop Dis. 2023 Oct 18;17(10):e0011341. doi: 10.1371/journal.pntd.0011341 (PMC10615262; doi:10.1371/journal.pntd.0011341)
Supplement: S1 Fig — (DOCX) [file pntd.0011341.s001.docx]

**S1 Fig:** Proportion of samples positive for *S.* Typhi according to distance from the river confluence in central Kathmandu.


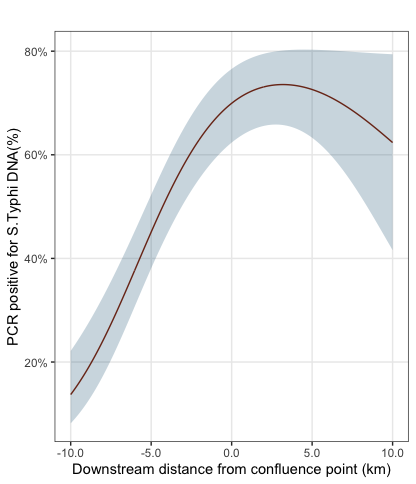


S1 Fig is a generalized additive model that plots the percentage of samples that are positive against by the distance samples are downstream from the river confluence in the center of Kathmandu. Samples 10 km upstream from the confluence are unlikely to be positive while closer to the confluence and up to 10km past the confluence more than 50% of samples are estimated to be positive. The estimated peak of sample positivity is at or a little beyond the river confluence area.
