# Supplementary material for: Environmental sampling for typhoidal Salmonellas in household and surface waters in Nepal identifies potential transmission pathways
Source: PLoS Negl Trop Dis. 2023 Oct 18;17(10):e0011341. doi: 10.1371/journal.pntd.0011341 (PMC10615262; doi:10.1371/journal.pntd.0011341)
Supplement: S1 Text — (DOCX) [file pntd.0011341.s002.docx]

**S1 Text: Limit of detection for drinking water assay**

**Limit of detection and spiking methods**

We first characterized the sensitivity of the laboratory methods we utilized by performing a limit of detection assay on our proposed extraction and amplification processes. We used linearized standard plasmid containing *S*. Typhi STY0201 (132bp) and *S.* Paratyphi SSPA7038 (105bp) target gene for these experiments. We conducted a set of 11-10-fold serial dilutions starting at a concentration of 114 ng/μl to 114 *×* 10^-11^ ng/μl for STY0201 and from 104 ng/μl to 104 *×* 10^-11^ ng/μl for SSPA7038. Each assay was performed in 4 replicates. The mean Ct value and associated numbers of detected DNA copies were recorded for each dilution in excel.

We additionally conducted a series of spiking experiments to understand the limit of detection in settings that more simulated the samples that we collected of drinking and river water. We prepared a standard 0.5 McFarland standard inoculum (approx. 1.5×10^^8^ CFU/ml) of Clinical *S*. Typhi strain in normal saline. We diluted the inoculum through a series of 8-10 fold dilutions with the most dilute solution having 1.5×10^0 CFU/ml *S*. Typhi. We then inoculated each dilution with 1.5×10^5^ CFU/ml *S*. Typhi to 1.5×10^0^ CFU/ml *S*. Typhi into separate 1L of commercially available mineral water bottles. These spiked water samples were mixed, then filtered through 0.45um Nalgene filter funnels. We performed DNA extraction on each spiked sample using Qiagen DNA PowerWater DNeasy Extraction Kit instructions and qPCR using the procedure available at (https://www.protocols.io/view/qpcr-protocol-cs9iwh4e).

**Limit of detection results**

In the limit of detection tests, the RT-PCR assay we used consistently detected samples down to the 10^-10^ dilution level, corresponding to a detection limit of 80 DNA copies per μl of sample. The average Ct value for *S*. Typhi DNA at this dilution was 35. At lower concentrations, the assay had lower sensitivity. For *S.* Paratyphi A, the minimum detectable dilution was also 10^-10^ which corresponds to 92 DNA copies per μl. The average Ct value for this dilution was 37. The minimum detectable dilution of spiked *S.* Typhi in 1 liter water samples was ~1500 colony forming units per liter (3.17 log cfu/L) which resulted in an average Ct value of 36 using qPCR in which each sample was processed in duplicate.
