# Supplementary material for: Environmental sampling for typhoidal Salmonellas in household and surface waters in Nepal identifies potential transmission pathways
Source: PLoS Negl Trop Dis. 2023 Oct 18;17(10):e0011341. doi: 10.1371/journal.pntd.0011341 (PMC10615262; doi:10.1371/journal.pntd.0011341)
Supplement: Attachment — Submitted filename: PLOS NTD Reponses to Reviewers Comments.docx [file pntd.0011341.s004.docx]

Dear Editor and Reviewers,

Thank you for your consideration of our manuscript and time in making thoughtful feedback and remarks on our manuscript. We have reviewed all of the editorial points and ensured that our manuscript now conforms to them. We have included our responses to each point brought up by reviewers and editors below.

Methods

*Comment 1: The sampling frame used for the drinking water sampling is unclear. The authors indicate that (lines 142 – 144): “We leveraged a population-based household survey conducted between 2017 and 2019 in Kathmandu and Kavrepalanchok districts, which included urban, peri-urban and rural communities[18]. From that survey, we randomly sampled households for drinking water.” The sampling process for the original survey should be elaborated on, along with the number of households which were sampled for the current study (and how this sample size was calculated) and the method of random sampling used.*

Response 1: We have added clarification to this section to address how households were selected. It now reads:

“For drinking water samples, we enrolled a geographically random, population-based sample from the SEAP hospitals catchment area which included urban, peri-urban and rural communities[18]. The catchment areas were split into grids and grid clusters were randomly selected to receive a healthcare utilization survey. Within these selected clusters we further randomly selected households with children (age 0–4 years, age 5–9 years, age 10–15 years, and age 16–25 years) for water sampling. The randomization process used has been previously published [18,19]. The locations of the households selected can be seen below (Figure 1).” (Lines 155-162)

*Comment 2: The sampling strategy for monthly river water samples appears to included 19 sites across at least 9 locations (indicated in lines 203 – 205 as being located at distances 1, 5 and 10 km upstream and downstream from the main river confluence point in the Kathmandu Valley and at three locations 5 km apart on the Punyamata river in the more rural Kavre District).*

*The authors indicated that they chose this sampling strategy because it allowed them to “sample from a range of environmental settings”. In the statistical methods the authors indicated they modelled the proportion of samples as a function of distance from the river confluence point using generalised additive models with logistic link functions (R package “mcgv”) (lines 267-268). The number of samples collected at each location or environmental setting is not clear making it difficult to determine if valid conclusions that can be drawn from any spatial modelling.*

Response 2: We have included clarification in the methods as to how many times each sampling site was sampled and the distribution of these locations. The section now reads:

“We collected monthly water samples between November 2019 and July 2021 from 19 environmental surveillance sites (ES sites). Among them, 16 sites were spread across 4 rivers upstream of the confluence point and a combined segment of these rivers, downstream the confluence point in Kathmandu Valley. Sites in each upstream river segment were located at distances 1, 5 and 10 km upstream of their confluence point with the main Bagmati river. We also sampled downstream points after the rivers combined including one location before the main confluence point and then three downstream points after all rivers combined at 1, 5, and 10 km downstream. In the more rural Kavre, samples were collected at 3 sites, 5 km apart along the Punyamata river with the middle site being directly downstream of the city of Banepa (Figure 3). We chose this sampling strategy because it allowed us to sample from a range of environmental settings, including locations upstream of the major city center where settlement was relatively sparse, concentrate most sample locations within the population dense areas, and sample at locations in agricultural areas downstream of Kathmandu and Banepa.” (Lines 209-221). We also made the following to address this above comment:At each location or site, we collected a “single” 50 ml water sample. Added to (Line 221.)

*Comment 3: The authors have used a observational study of river usage at each 19 river water sampling sites to describe some of the possible sources of river water contamination. Although it is indicated that a mobile application was used to collected data on abiotic factors (line 215-216), the frequency of collection of this data at each site is not indicated. It is also not clear whether a standardised tool was used, and whether staff were trained in data collection to minimise bias.*

Response 3: We have added clarification on the frequency of collection of abiotic factors and questions around river contact:

“At each sampling location and timepoint, team members used a standardized survey to record information on abiotic factors that may affect bacterial input or survival in the water. The survey included questions on including observation of sewage pipes draining into the water, fecal matter near the site, and human / animal presence and activities at the sampling point. All information was entered into the mobile application Redcap v5.19.15(Vanderbilt University, Nashville,TN) Team members also used a probe to collect readings of the water pH, temperature, and oxygen reduction potential (Apera Instruments, Columbus, OH, AI311) . Our field sampling protocol can be found online at…” (Lines 226 - 235)

*Comment 4: Methods on statistical analyses (line 258-271) should be revised include:*

*- details of basic analysis to determine differences in the frequency of S. Typhi and S. Paratyphi sample positivity, including statistical tests and the level of significance used.statistical*

Response 4: We appreciate your pointing out the lack of clarity in how we generated these statistics. We have provided more clarification in the methods section about how the different statistics were calculated.

For drinking water samples:

“We assessed differences in drinking water sourcing between Kathmandu and Kavre using chi square tests using p<0.05 as a measure of statistical significance” (Lines 276-278)

For River water:

“We constructed generalized linear mixed effect models, employing the function glmer() from the “lme4” package in R to test for differences between sample positivity based on seasonality (monsoon vs. dry), region (Kathmandu vs. Kavre), and segments of the Kathmandu Valley rivers (upstream, city center, and downstream). Sample site id was included as a random effect in each model. Statistical significance was assessed at a 95% level using a Wald test” (Lines 285-290)

*Comment 5: Detail of how trend lines were fitted to Figures 2B and 2C*

Response 5: We have included additional details as to how these regressions were made. It now reads: “We used locally weighted regression (loess) to create smooth lines of rainfall by month (Figure 2B) and to relate river water positivity and month of the year (2C). For water sample detection (2C), we created separate smooth lines for January – July and September – December due to there being a data gap in August.” (Lines 280-284)

Reviewer #2: The objectives are clear

The study design is appropriate

The population is clearly described

The sample size is sufficient

There are no ethical concerns

Reviewer #3: (No Response)

--------------------

Results

Reviewer #1:

*Comment 6: The results section begins by presenting the findings of the limit of detection tests (lines 273-281). The relevance of these results to the overall study hypothesis is not clear. It is suggested that this is included as supplementary material.*

Response 6: We agree with the reviewer on this point and had included the limit of detection data for clarification to readers on how well the tests worked but now have shifted the information on the methods and results of the assay testing methods and results to a supplementary appendix.

The text now reads: “We performed a series of limit of detection and spiking experiments to characterize the sensitivity of the laboratory protocols we used for the drinking water assay. More information on these experiments and their results can be found in Appendix 1.” (Lines 189-191)

*Comment 7: The authors variably stratify the results for river water sample analysis by month, season, location (Kathmandu Valley and Kavre), sampling site, distance from confluence or by population density, as well as describing overall frequency of serovar detection in all samples. Drinking water sample results describes the source of household water supply and the number of sample where serovars were detected by location (Kathmandu Valley and Kavre) and water source. In both instances, data is presented in the text as counts by group (sometimes with a denominator, proportions and p values for tests of significance). The clarity for the reader of the data in this results section would be greatly improved through the presentation of data in a table/s. The text of the results section can then be reviewed and minimized by referring to these data tables.*

Response 7: We appreciate this suggestion. We have added four tables to the results section (Table 1, Table 2, Table 3), each stratified by the variables that we describe in the text.

Table 1: Contains the number of household water samples and their associated positivity (Line 308)

Table 2: Contains river water sample results and associated viability and percent positivity, stratified by seasonality, distance on river and region (Line 338)

Table 3: Contains the number of sites at which human interaction with rivers occurred and times when these observations correspond with positive samples. (Line 415)

*Comment 8: River water testing data is somewhat difficult to interpret as it includes both outcomes of methodological validation (the role of the enrichment step and the discussion of pathogen viability) as well as aggregate and stratified water contamination data. It is suggested that the authors use subheadings and present the data tables to help the reader navigate.*

Response 8: As suggested, we have added sub-headers throughout the results section as well as added the new tables described above.

*Comment 9: It is unclear how important pathogen viability is to discuss in the context of study hypothesis.*

Response 9: We have added two sentences in the introduction (starting at line 142) describing the importance of viability to our study hypothesis. It reads:

“Diagnosis of enteric fever in humans is done through culture based methods, but culturing enteric fever causing *Salmonellae* from the environment is notoriously difficult, as the bacterium are hypothesized to enter a viable but not culturable state in surface water [8,17,18]. PCR techniques are commonly used to determine if *S.* Typhi or *S.* Paratyphi DNA is present in environmental samples, but detecting DNA may not indicate the presence of viable bacteria [19–22]. For *Yersinia pestis* and *Bacillis anthrasis,* researchers use a liquid culture enrichment step and duplicate DNA extractions to test for pathogen viability. We have adapted this method for *S.* Typhi and *S.* Paratyphi A[23,24]. (Line 142-150)

*Comment 10: A number of approaches are detailed in the methodology for which no results are presented in the main manuscript text. The authors are recommended to correct the mismatch in this content:*

*Comment 10a: Lines 160 – 163: household questionnaires on drinking water purification*

Response 10a: As suggested, we have now elaborated on this relationship in the text by stating that:

“38% of households that responded (n=289/370) reported treating their water before drinking it. Three samples that tested positive for *S.* Typhi came from households that reported treating their water, two from households that did not treat water and two from households that had not answered the question.” (Lines 323-327)

*Comment 10b: Lines 214 – 220: collection of abiotic factors that may affect bacterial input or survival (water pH, temperature, oxygen reduction potential)*

Response 10b: We have now included a sentence about this in the results section: “We did not observe any association between sample positivity and pH, water temperature, or oxygen reduction potential.” (Lines 350-352)

*Comment 10c: Lines 267 – 268: Modelling of the proportion of samples as a function of distance from the river confluence point. No reference has been made in the main results text of the manuscript to the data presented the supplementary information annex.*

Response 10c: We have updated the text to now include a note describing the odds of the downstream points results of the generalized attentive model and how it augments the results otherwise described. It now reads

“Our mixed effects model shows that the odds of a sample from the city center containing *S.* Typhi DNA was 11.3 times more likely (95% CI: 4.3, 33.1) and from a downstream site 14.5 times more likely (95% CI: 4.4-50.8) than the odds of an upstream sample testing positive for *S.* Typhi. A generalized additive model (Supplementary Figure 1) also shows increasing rates of positivity the further downstream samples are taken, except for a slight tapering of sample positivity for *S.* Typhi at the point 10 km downstream of Kathmandu city limits.” (Line 383-390)

*Comment 11: Figure titles should be amended to include details of place and time of the study, and sample size where relevant (eg Figure 1: Drinking Water Sampling Locations, Kathmandu Valley and Kavre district, February to October 2019.)*

Response 11: We have added the additional metadata requested to the Figure titles. We also have included a supplementary data table uploaded to dryad with the data tables directly used to create each figure.

*Comment 12: Figure 1 is unclear as includes labels for Kathmandu, Dhulikhel and Banepa, while the figure caption refers to Kavrepalanchok. The authors should avoid to include methodology of sampling in the figure caption in the results section. It is suggested that Figure 1 could be moved instead to the method section.*

Response 12: We have changed the text on the figure to now read Kavre instead of Banepa and Dhulikhel and have changed the wording in the caption to Kavre instead of Kavrepalanchok. We included in the abbreviations on the first page Kavre corresponds to the district’s longer name Kavrepalanchok. We have moved the figure to the methods section as recommended to give readers more context as to where the samples are collected from. Line 163

Updated figure text: **“Figure 1: Drinking Water Sampling Locations, Kathmandu Valley and Kavre district, February to October 2019**

The location of the households from which our team collected the 370 drinking water samples reported on in this study. The aggregation of clusters of points in the Kavre district reflects the two peri-urban areas of the region, Banepa to the left and Dhulikhel, to the right.”

(Lines 166-168)

*Comment 13: The title and caption for Figure 2 should include a sample size for each month as this relates to monthly rain (B) and river water sampling (C). The caption for Figure 2A should describe what statistic is represented by the error bars in this figure. As river water samples were not collected in August, the trend line provided in part C, should have a break at this point.*

Response 13: We have updated the figure caption but were unable to update the title as each different section of the graph has different sample sizes. The caption now reads:

“A. The mean number of *E. coli* coliforms present per mL of sample by month from July 2020 to July 2021. There were 6 coliform samples collected in each month except for July when coliform samples were taken on the first and third week of the month for a total of 11 samples. August was missed in both years of sampling due to COVID-19 lockdowns. B. Monthly rainfall from 2015-2019 taken from Kathmandu Airport and Kavre government meteorological stations (n=6 for each location each month) C. The proportion of river water samples PCR positive for *Salmonella* Typhi or Paratyphi A by month in 380 samples from July 2019 to May 2021.” (Lines 364-370)

We also updated the trend line in figure 2C so that it has a break through the month of August.

Comment 14: Figure 3 would benefit from presentation in a large format and better markers on each map for distance and orientation of upstream and downstream locations.

Response 14: We have increased the size of Figure 3, added the timeline of sample collection in the figure title, added arrows to indicate the direction of water flow and updated the caption of the figure to make it more clear. The caption now reads:

“The 19 sampling points were each sampled 20 times at approximately a monthly interval. The proportion positive for *Salmonella* Typhi (grid A) and *Salmonella* Paratyphi A (grid B) are indicated by color of the sampling point. The size of the sampling point indicates the number of people estimated to live upstream of that point which was calculated using ArcGIS Hydrosheds. The population density in units of people per 100m^2^ is indicated in pink shading. Water flow direction goes from North to South as indicated by the black arrows in the upper pane..”(Line 399-404)

*Comment 15: The authors should consider including data on observations on river usage (line 362) in a table, possibly stratified by observations at collection sites in Kathmandu Valley and Kavre if this is relevant. This data is limited in that it can only give an impression of sources of contamination – the authors should avoid to give this data too much emphasis.*

We have now included the human interactions with rivers data in a table (Table 3) and added a sentence to the text about the distribution of these observations between Kathmandu and Kavre. It reads, “Human interactions with rivers mainly occurred within the Kathmandu Valley. People were observed walking, washing clothes and bathing in only the most upstream point in the Kavre region” (Line 422-424)

*Comment 16: The results should be carefully reviewed for consistency and accuracy – for example the number of household drinking water samples collected is variably reported as 370 (line 283), 400 (line 289).*

We appreciate the reviewer identifying this issue. We have corrected the erroneous 400 to now reads “The location of the households from which our team collected the 370 drinking water samples reported on in this study.” We have checked the rest of the numbers in the results again. (Line 166)

Reviewer #2: The analysis matches the analysis plan

Results are clearly presented

The quality of the figures and images is of good quality and sufficient

Reviewer #3: (No Response)

--------------------

Conclusions

*Comment 17: Reviewer #1: The presentation of the conclusions on household drinking water contamination should be reviewed. The authors mostly re-state the results. It is not clear where the main conclusions from the current study and the comparison to findings in the literature are separated. For example in the following text it is difficult to distinguish the what information is contributed by reference 33 and by the current study – lines 407-410 “In our study, less than 50% of households sampled from Kathmandu or the Kavre region (154/370) used municipal water as their main drinking water source although slightly more households used municipal water from Kavre than did in Kathmandu[33].” The authors should discuss the findings in context of literature relating to different transmission pathways for S. Typhi compared to S. Paratyphi.*

Response 17: We have added information in the introduction clarifying how different transmission pathways between the species may be facilitated by infectious dose into the introduction. This amendment reads. “Mounting evidence shows *S.* Paratyphi A may follow different transmission dynamics than *S.* Typhi[17,18]. Specifically, a case control study from the Kathmandu valley showed *S.* Paratyphi was likely transmitted by food while S. Typhi transmission was associated with poor quality water usage [18].” (Line 124-127)

*Comment 18: Conclusions on the role of contamination of vegetables from river water in the transmission pathway of S. Typhi and S. Paratyphi should not be overstated (line 432 – 441). Food sampling at the point of consumption and epidemiological studies to investigate the exposure to contaminated food in infected individuals provide better strength of evidence for associations.*

Response 18: We agree that further studies would be needed to determine whether contamination of vegetables or other agricultural products plays an important role in typhoidal *Salmonella* transmission in this context. We have revised our discussion of this to acknowledge this:

“Despite the presence of *S.* Typhi and Paratyphi DNA contamination in samples from sites with human interactions and vegetable washing, our study does not provide any evidence of association of contaminated river water or produce with human typhoid infections. Sampling of food at the point of consumption, and epidemiological studies assessing exposure to potentially contaminated food sources and risk of enteric fever, should be undertaken to better investigate this potential pathway.” (Line 539-544)

*Comment 19: Several of the limitations of the study discussed by the authors relate to the methodology used to analyse water samples. The authors should reflect on next steps and information which should be collected to better understand transmission of S. Typhi and S. Paratyphi. Environmental sampling should not be considered in isolation – reports of human cases of enteric fever and well designed epidemiological studies may better identify sites and risk factors for exposure and transmission.*

Response 19: We have added additional discussion to the limitations section on how environmental detection work could be augmented with additional epidemiologic data in the future to better understand pathways of transmission in this area. It now reads:

“Sampling of food at the point of consumption, and epidemiological studies assessing exposure to potentially contaminated food sources and risk of enteric fever, should be undertaken to better investigate this potential pathway. Moreover, testing irrigation water in fields alongside the contaminated sections of river could help us understand if contaminated surface waters are interacting with nearby fields. The transmission pathways of *S.* Typhi and *S.* Paratyphi A are complex and multifaceted. Continuing environmental surveillance after the new typhoid vaccine was introduced could additionally help understand if the vaccine influences the prevalence of enteric fever causing *Salmonellas* in downstream water supplies.” (Lines 542-550)

*Comment 20: Reviewer #2: The conclusions are supported by the data presented and the limitations are clearly highlighted.*

*The authors describe the need for further investigations to link positivity of river water samples to risk of exposure to typhoidal pathogens and disease*

Reviewer #3: (No Response)

--------------------

Editorial and Data Presentation Modifications?

*Comment 21: Reviewer #1: The focus of this study is the understanding of environmental (water) sources of Salmonella enterica serovars Typhi and Paratyphi with the view to identifying possible pathways of transmission in the Kathmandu Valley and rural Kavre district of Nepal. The author introduce the use of vaccines and antibiotic treatment and their partial effectiveness in controlling transmission, as a means of justifying why environmental sampling is also necessary (lines 97-107). The authors should consider consolidating the information on vaccines as this should be provided for context of research focus only and doesn’t relate to conclusions of the research. The authors use an example from Bangladesh to describe effectiveness of TCV mass vaccination campaign. Given the location of the research, it would be more appropriate to discuss vaccination against enteric fever in Nepal. The authors could consider the study by Shayka et al (The Lancet Global Health. 2021;9(11):e1561-1568)*

*and acknowledge that TCV has recently been introduced in Nepal (*[*https://www.unicef.org/nepal/press-releases/nepal-introduces-typhoid-vaccine-routine-immunisation-across-country*](https://www.unicef.org/nepal/press-releases/nepal-introduces-typhoid-vaccine-routine-immunisation-across-country)*).*

Response 21: We have shortened the section on antibiotics and vaccines and changed the numbers and references around vaccine effectiveness to focus on Nepal, not Bangladesh. The section now reads:

“The World Health Organization (WHO) recently prequalified two typhoid conjugate vaccines (TCV), one of which was shown to be effective in reducing typhoid incidence in three randomized trials[7–9]. In a Phase 3 trial of TCV conducted in Nepal, vaccine recipients had an typhoid fever incidence of 79 cases per 100,000 person-years, indicating that even effective vaccines alone cannot end transmission [7]” (Line 100-105)

*Comment 22: The introduction of the paper only briefly focuses on the epidemiology of typhoid fever transmission in Nepal (lines 94-96, with a small mention of the impact on typhoid case numbers resulting from changes in aquifer levels and after the Kathmandu Valley earthquake (lines 121 and 123)). It is recommended that the manuscript includes more information on the geographical and temporal distribution of cases in Nepal and specifically in the Kathmandu Valley and Kavre district (for example Andrews et al The Journal of Infectious Diseases. 2017;jix221:S1-S8. and Baker S, et al. Open Biol 2011; 1:110008.) especially as these relate to the context and objectives of the current study. Data from references 9 and 39 could also be included here.*

Response 22: We have added a additional information on the spatial dynamics of typhoid fever in Nepal and the Kathmandu valley more specifically. The section now reads:

“Within Kathmandu and Kavre Districts of Nepal, enteric fever incidence rates vary widely by ward, a sub-administrative unit, with S. Typhi and S. Paratyphi A infections clustering nearby one another in low lying areas[12,13]. Drinking water has long been recognized as an important route of *Salmonella* Typhi and Paratyphi A[13]. Municipal water in the Nepal’s southern state of Bharatpur was implicated in the largest single source outbreak of typhoid fever recorded to date[14]. S. Typhi has been isolated from municipal taps from the Kathmandu Valley on multiple occasions [9,12,15]. Work by Karkey et al. found high levels *S.* Typhi (77% sample positivity) and *S.* Paratyphi A (70%) DNA in water collected from public drinking water spouts [9]. In the twelve years since their samples were collected, lowering aquifer levels and a large earthquake rendered many of the aquifers they tested inoperable yet enteric fever cases in the Kathmandu Valley have remained high [3,9,16]. Mounting evidence shows *S.* Paratyphi A may follow different transmission dynamics than *S.* Typhi[17,18]. Specifically, a case control study from the Kathmandu valley showed *S.* Paratyphi was likely transmitted by food while *S.* Typhi transmission was associated with poor quality water usage [18]. A recent case-control study from Malawi found use of river water for cooking and cleaning to be the strongest individual risk factor for typhoid fever [19]. In the Kathmandu Valley, *S.* Paratyphi A infection clusters are more geographically dispersed than those of *S.* Typhi clusters and are most often found in areas close to the Bagmati river [12].” (Lines 112-131)

*Comment 23: The title of this paper and the body of the manuscript, including the conclusions frequently refers to the activities of environmental water sampling and testing described in the paper as “environmental surveillance”. While river water samples were collected at designate locations approximately monthly between November 2019 and July 2021, this manuscript should be careful that it does not overstate the presented research as surveillance. A previous paper by some of the co-authors very nicely prepares a rationale for environmental surveillance as a tool for identifying typhoid transmission (Andrews et al CID, 2020:71 (Suppl 2): listed as reference 20 in this manuscript). The present manuscript would benefit from similar reflections from these co-authors.*

Response 23: We hear the reviewer’s point that this research study consisted of environmental sampling and was not the construction of an environmental surveillance system. We have updated the paper title and sub-headings changing “Environmental Surveillance” to “Environmental Sampling” and updated the text to match

*Comment 24: As a separate note, reference 20 (which appears on line 193) is incorrectly attributed to primer and probe sequences of Nga et al.*

Response 24: We had included two references at the end of this sentence to show the primer codes Nga et al. reference 21 and and included reference 20 to reference something said earlier in the sentence. We have now broken the sentence into 2 to aid in ease of reference. It now reads:

“After extraction, we performed real-time PCR (RT-PCR) to detect *S.* Typhi and *S.* Paratyphi A DNA using previously published primers and probes from Nga et al.” (Lines 199-201)

*Comment 25: This manuscript refers often to “typhoid fever” or “typhoidal Salmonellas” or “typhoid” when referring to these two serotype drinking water and “typhoid fever exposure”, dominant mechanisms of (typhoid) transmission or “Salmonella exposure” (lines 112 to 117). The authors are recommended to review the nomenclature used in their manuscript – and should take care when generalising disease due to paratyphoid and typhoid together especially as some studies suggest the risk factors for the development of enteric fever due to S. Typhi and S. Paratyphi differ (Karkey A et al PLoS Negl Trop Dis. 2013;7(8):e2391 and Levantesi, C. et al Food Res. Int., 45 (2012), pp. 587-602). The expertise of some of the senior co-authors of this manuscript (including Associate Professor Andrews) could be drawn on to improve the clarity around these descriptions.*

Response 25: We have corrected the nomenclature throughout the manuscript. We appreciate the reviewer linking us to these pieces and have included more in the introduction on the differences in *S.* Typhi and *S.* Paratyphi transmission. It reads:

“Mounting evidence shows *S.* Paratyphi A may follow different transmission dynamics than *S.* Typhi[17,18]. Specifically, a case control study from the Kathmandu valley showed *S.* Paratyphi was likely transmitted by food while *S.* Typhi transmission was associated with poor quality water usage” (Lines 124-127)

*Comment 26: Given the level of methodological detail included in this manuscript, the rationale for use of the selected sample analysis approaches for household drinking water and river water samples has not been included in the introduction, with only a small discussion of the included about the limitations associated with the use of enrichment steps (lines 469 – 481). More background should be provided on gold standard testing approaches for S. Typhi and S. Paratyphi in water samples, and why qPCR and enrichment methods were used.*

Response 26: We have added additional information about gold standard approaches for testing for *S. Typhi and S.* Paratyphi in the introduction and why this method was chosen.

It now reads” “Diagnosis of enteric fever in humans is done through culture based methods, but culturing enteric fever causing *Salmonellas* from the environment is notoriously difficult, as the bacterium are hypothesized to enter a viable but not culturable state in surface water [8,21,22]. PCR techniques are commonly used to determine if *S.* Typhi or *S.* Paratyphi DNA is present in environmental samples, but detecting DNA may not indicate the presence of viable bacteria [23–26]. For *Yersinia pestis* and *Bacillis anthrasis,* researchers use a liquid culture enrichment step and duplicate DNA extractions to test for pathogen viability. We have adapted this method for *S.* Typhi and *S.* Paratyphi A [27,28]. We test drinking water and surface water (river) samples from both the Kathmandu and Kavre Districts and record human river interactions to understand the potential role of other waterborne transmission routes of typhoid fever in this area.” (Lines 142-147)

*Comment 27: Other more general edits are also recommended:*

*-	The text is sometimes clumsy when referring to and describing other published work. For example (lines 116-121) has a long description of work which appears in reference [9]. Initial the authors mention “previous work” without a reference. The manuscript directly quotes the conclusions of Karkey and colleagues, without mentioning the authors by name (“they found”…. “which led them to conclude”). For brevity and clarity it is suggested that structure of these sentences should be reviewed.*

Response 27: We have altered these sentences to aid in clarity and readability of this section of the manuscript. It now reads:

“Work by Karkey et al. found high levels *S.* Typhi (77% sample positivity) and *S.* Paratyphi A (70%) DNA in water collected from public drinking water spouts [8].” (Lines 119-121)

*Comment 28: Line 117 – referring to Salmonella exposure is too general: a species (Salmonella enterica) or serovar/s (S. Typhi / S. Paratyphi) must be specified.*

Response 28: We now refer to *S.* Typhi and *S.* Paratyphi A in this sentence, as follows: .

“ Mounting evidence shows *S.* Paratyphi A may follow different transmission dynamics than *S.* Typhi[17,18]. Specifically, a case control study from the Kathmandu valley showed *S.* Paratyphi was likely transmitted by food while *S.* Typhi transmission was associated with poor quality water usage [18].” Lines 124-127

*Comment 29: Refer to household drinking water or drinking water sampled from households, not “drinking water” (review throughout document)*

Response 29: As suggested, we have changed all mentions of drinking water in the document to drinking water sampled from households or “household drinking water”..

*Comment 30: Kavre and Kavrepalanchok are used interchangeably – a consistent name should be used*

Response 30: We have changed all instances of the word Kavrepalchok being used to just Kavre and have added this note to the abbreviations list on the first page.

Comment 31: Notes under Figure 2 mention Coronavirus Lockdowns. It is more correct to refer to COVID-19 lockdowns or COVID-19 pandemic restrictions.

Response 31: We have changed this to COVID-19 lockdowns. (Line 363)

Comment 32: References to websites within the text should include an access date.

Response 32: We have added access dates to all links included in the text.

*Comment 33: LINE 65: Please delete ‘s’ …….srepresent*

Response 33: Thank you. We have removed this sentence in rewording the author summary.

*Comment 34: LINE 90: Should be ….’over 100,000 deaths’*

Response 34: We have corrected this sentence, which now reads:

“*Salmonella* Typhi and Paratyphi A which cause typhoid and paratyphoid fever respectively infect approximately 10 million people and cause over 100,000 deaths annually.” Line 91-93

*Comment 35: LINE 97 to 99: This statement is confusing .......Ty21a is a whole cell inactivated vaccine orally administered, while the Vi is a subunit vaccine intramuscularly administered*

Response 35: Thank you for correcting the administration pathways. We have removed the pathways of vaccine administration because it is extraneous to the work of environmental surveillance and just included a note on the new vaccine. Line 96

*Comment 36: LINE 106: ‘also’ is repeated……. Ending transmission also must also*

*address environmental and political issues.*

Response 36: We appreciate the reviewer identifying this redundant word. The first also has been removed. It now reads “Ending transmission must also

address environmental and political issues.” Line 100

*Comment 37: LINE 113: Should be ……’different times’*

Response 37: This sentence was removed.

*Comment 38: LINE 116: Add spacing beginning of sentence ……” In Nepal, previous work implicated…….”*

Response 38: This sentence has been eliminated.

*Comment 39: LINE 186: How long were the holidays and does this affect the results?*

Response 39: Upon consultation with the team we removed the wording about holidays because they had ensured collection periods were outside of the holiday weeks of Tihar and Dashain, and so there were not extended periods of the samples being left at 4 deg C.

Comment 40: LINE 205 to 206: Please rewrite this sentence to clearly indicate the characteristics of the locations included

Response 40: We have made this more clear in the text as follows:

“Each day of collection, research staff collected a field negative control at the final sampling site by pouring sterilized distilled water from a bottle into a Falcon tube and placing it in the sample bag for transport back to the laboratory.” (Line 224-226)

*Comment 41: LINE 218: Should ‘point’*

Response 41: We regret this typo. The “i” has been included so it now reads “point”

*Comment 42: LINE 229: To be exact would this be 44.8 mL*

Response 42: We have updated this to now read “We processed the remaining 44.8 mL” Line 244

*Comment 43: LINE 269: Should be…… ‘The code used…….’*

Response 43: Thanks - now reads “the code used…”

*Comment 44: LINE 277 onwards: please italicize ‘S’ for Salmonella*

Response 44: We have gone through the entire documents Italicizing all *S. ‘s* and using the phrasing Paratyphi A instead of just Paratyphi

*Comment 45: LINE 399: Add space beginning of new sentence*

Response 45: We added a period and space at the start of the sentence.

*Comment 46: LINE 401: There is a missing word……is it ‘surveyed?’*

Response 46: We have added the missing word ‘surveyed’. The sentence now reads:

“When we sampled drinking water from households in the Kathmandu Valley and Kavre Districts, we found that no households reported using these spouts for drinking water procurement,” (Line 445-447)

*Comment 47: LINE 421: There is a missing word after ‘sporadic’*

Response 47: We changed sporadic to “intermittent flow” to describe the noncontinuous flow of municipal water. (Line 464)

*Comment 48: LINE 423 to 424: Does it matter how further downstream a location is? Does the topography of the section of the river being sampled matter and corresponding speed of the waters going downstream?*

Response 48: We investigated this relationship between distance and positivity through a generalized additive model. We now report this result as follows::

“The generalized additive model (Supplementary Figure 1) also shows increasing rates of positivity the further downstream samples are taken, except for a slight tapering of sample positivity for *S.* Typhi at the point 10 km downstream of Kathmandu city limits.” (Line 386-389)

We did not have a measurement of flow rate of the water to allow us to measure the association of flow to the rate of positivity. Anecdotally flow rate increased during the monsoon season when rivers were most full of water, but the flow rate across the different sampling points in and below the Kathmandu valley does not seem to vary greatly at the same time point.

*Comment 49: LINE 418 to 421: for consistency you can maintain use of numbers to present figures, within the sentence.*

Response 49: As suggested, we have revised as follows: “5 of the positive samples” (Line 463)

Reviewer #3: (No Response)

--------------------

Summary and General Comments

*Comment 50: Reviewer #1: The overall objective of the study is stated as: “This study seeks to understand the extent to which different water sources are contaminated with typhoid fever causing bacteria S.Typhi and .Paratyphi A across the urban Kathmandu Valley and more rural Kavre district of Nepal.”*

*The content of this manuscript represents a large amount of work, including methodological development, however care should be taken to avoid publishing all of an academic endeavour as a single body of work while balancing with sufficient content to adequately test a hypothesis. The authors are encouraged to edit and refine the manuscript to clearly focus the presentation of the methodology and results on the main objective of the study and generate a clear narrative to arrive at the conclusions (including limitations and next steps).*

Response 50: We have streamlined the flow of the manuscript by moving all of the discussion around the limit of detection methods and results to the supplementary table, made the background section more Nepal specific, made clearer references to figures and added tables of results to increase the ease of understanding results. We have also edited the discussion and conclusions to improve the flow and messaging of the piece.

Comment 51: Reviewer #2: I would like to commend the authors for their research and study findings, which add to the mounting evidence that the pathways leading to typhoidal disease can be influenced by not only drinking water but also by using water to wash and clean fruits and vegetables.

I have found areas in the manuscript that need to be clarified and corrected for the reader's benefit.

Response 51: We thank this reviewer for their editorial comments suggested earlier and have made these edits. We have included responses to each of their comments above

*Comment 52: Reviewer #3: These study findings are very important to disseminate to prevent the transmission of S. Typhi by implementing effective preventive strategies.*

*Minor comments are as follows:*

*It will be good if the authors can add any surveillance data to show the positivity rate of typhoid infection among people who used the contaminated water during the study time.*

Response 52: We thank the reviewer for this suggestion. While we do not have data on typhoid positivity according to specific contaminated water exposures, we have generated data on typhoid incidence in Kathmandu and Kavre. We have added the following to the manuscript to report these data:

“A recent population-based study reported a high incidence of typhoid fever in Kathmandu (330 cases per 100,000 person-years) and Kavre (268 cases per 100,000 person-years).” (Lines 96-98)

*Comment 53: Moreover, the authors have only observed that people wash their vegetables with river water, but they did not take any history if they have consumed these vegetables cooked or without cooking. History of taking outside foods is also important.*

Response 53: We agree with the reviewer that this is a limitation of our study and merits further study which we have noted in the limitations of this piece.

*Comment 54: The authors should mention the name of model used for the analysis in the statistical analyses section. What are the covariates used for the adjustment should be mentioned here? Have they used season as a confounding variable?*

Response 54: We now provide the names of all models used in the manuscript, as follows:

Chi square

Generalized linear mixed effect

Generalized additive

Locally weighted regression

A longer explanation of the changes made are included in the responses to reviewers in the the methods section of the responses to reviewer comments above.

*Comment 55: The authors should make all “S” and “salmonella” italic throughout the manuscript in case of S. Typhi and S. Paratyphi.*

Response 55: We have gone through the manuscript and italicized all *Salmonellas* throughout the paper.

Comment 56: The authors should check the manuscript thoroughly and rewrite it where need to improve the English of this manuscript.

Response 56: We have reviewed the manuscript to check for grammatical correctness and have modified accordingly.

*Comment 57: Figure Files:*

*While revising your submission, please upload your figure files to the Preflight Analysis and Conversion Engine (PACE) digital diagnostic tool,* [*https://pacev2.apexcovantage.com*](https://pacev2.apexcovantage.com/)*. PACE helps ensure that figures meet PLOS requirements. To use PACE, you must first register as a user. Then, login and navigate to the UPLOAD tab, where you will find detailed instructions on how to use the tool. If you encounter any issues or have any questions when using PACE, please email us at* [*figures@plos.org*](mailto:figures@plos.org)*.*

Response 57: We have uploaded all figures included in this text to Pace

**Comment 58:**

***Data Requirements:***

*Please note that, as a condition of publication, PLOS' data policy requires that you make available all data used to draw the conclusions outlined in your manuscript. Data must be deposited in an appropriate repository, included within the body of the manuscript, or uploaded as supporting information. This includes all numerical values that were used to generate graphs, histograms etc.. For an example see here:* [*http://www.plosbiology.org/article/info%3Adoi%2F10.1371%2Fjournal.pbio.1001908#s5*](http://www.plosbiology.org/article/info%3Adoi%2F10.1371%2Fjournal.pbio.1001908#s5)*.*

Response 58: We have included all data from this paper into Dryad. We have also created an additional dataset based on the example above that includes the numerical values used to render each figure presented in the text. The link to the repository is here (DOI: 10.6078/D1ZD84)

Comment 59:

*Reproducibility:*

*To enhance the reproducibility of your results, we recommend that you deposit your laboratory protocols in protocols.io, where a protocol can be assigned its own identifier (DOI) such that it can be cited independently in the future. Additionally, PLOS ONE offers an option to publish peer-reviewed clinical study protocols. Read more information on sharing protocols at* [*https://plos.org/protocols?utm_medium=editorial-email&utm_source=authorletters&utm_campaign=protocols*](https://plos.org/protocols?utm_medium=editorial-email&utm_source=authorletters&utm_campaign=protocols)

Response 59: We have uploaded all protocols used to protocols.io and have included the links to the protocols within the manuscript when referencing these protocols.
